# Triiodothyronine or Antioxidants Block the Inhibitory Effects of BDE-47 and BDE-49 on Axonal Growth in Rat Hippocampal Neuron-Glia Co-Cultures

**DOI:** 10.3390/toxics10020092

**Published:** 2022-02-18

**Authors:** Hao Chen, Rhianna K. Carty, Adrienne C. Bautista, Keri A. Hayakawa, Pamela J. Lein

**Affiliations:** Department of Molecular Biosciences, University of California, Davis, CA 95616, USA; hachen@ionisph.com (H.C.); rhianna.k.carty@gmail.com (R.K.C.); abcashion@ucdavis.edu (A.C.B.); kahayakawa@ucdavis.edu (K.A.H.)

**Keywords:** axonal growth, developmental neurotoxicity, neuronal morphogenesis, PBDE, reactive oxygen species, thyroid hormone

## Abstract

We previously demonstrated that polybrominated diphenyl ethers (PBDEs) inhibit the growth of axons in primary rat hippocampal neurons. Here, we test the hypothesis that PBDE effects on axonal morphogenesis are mediated by thyroid hormone and/or reactive oxygen species (ROS)-dependent mechanisms. Axonal growth and ROS were quantified in primary neuronal-glial co-cultures dissociated from neonatal rat hippocampi exposed to nM concentrations of BDE-47 or BDE-49 in the absence or presence of triiodothyronine (T3; 3–30 nM), N-acetyl-cysteine (NAC; 100 µM), or α-tocopherol (100 µM). Co-exposure to T3 or either antioxidant prevented inhibition of axonal growth in hippocampal cultures exposed to BDE-47 or BDE-49. T3 supplementation in cultures not exposed to PBDEs did not alter axonal growth. T3 did, however, prevent PBDE-induced ROS generation and alterations in mitochondrial metabolism. Collectively, our data indicate that PBDEs inhibit axonal growth via ROS-dependent mechanisms, and that T3 protects axonal growth by inhibiting PBDE-induced ROS. These observations suggest that co-exposure to endocrine disruptors that decrease TH signaling in the brain may increase vulnerability to the adverse effects of developmental PBDE exposure on axonal morphogenesis.

## 1. Introduction

The brominated flame retardants, polybrominated diphenyl ethers (PBDEs), are considered to be likely environmental risk factors for neurodevelopmental disorders [1,2,3,4]. Epidemiologic studies have identified a negative association between developmental exposure to PBDEs and executive function, motor behavior, and attention in infants and children [5,6,7,8,9,10,11,12]. These findings are of significant public health concern given the documented widespread human exposure to PBDEs with significantly higher body burdens in infants and toddlers relative to adults [13,14]. However, there remains significant uncertainty regarding the underlying mechanism(s) by which PBDEs interfere with neurodevelopment.

It has been hypothesized that PBDE developmental neurotoxicity reflects altered patterns of neuronal connectivity [12,15,16]. A critical determinant of the patterns of connections formed between neurons during development is axonal morphology. Interference with temporal and/or spatial aspects of axonal morphogenesis has been shown to cause functional deficits in experimental models [17,18,19]. Moreover, altered patterns of axonal growth are implicated in the pathogenesis of various neurodevelopmental disorders [20,21]. Recently, we demonstrated that BDE-47, a PBDE congener that is highly abundant in human tissues, and BDE-49, an understudied PBDE congener with levels comparable to BDE-47 in gestational tissues of women living in southeast Michigan [22], inhibited axonal growth in primary hippocampal neuron-glia co-cultures, in part by delaying neuronal polarization [23].

BDE-47 and BDE-49 effects on axonal growth in primary hippocampal neurons were prevented by pharmacological blockade of ryanodine receptors (RyR) or siRNA knockdown of RyR, implicating RyR-dependent mechanisms in PBDE developmental neurotoxicity [23]. However, an unexpected finding from our previous studies was that the axon inhibitory effects of BDE-47 and BDE-49 exhibited comparable concentration-effect relationships despite significant differences in their potency at the RyR [24]. This observation raised the possibility that the RyR is not the primary molecular target but rather a downstream effector in the adverse outcome pathway (AOP) linking PBDEs to axonal growth inhibition. PBDEs have been shown to interfere with thyroid hormone (TH) signaling and to cause oxidative stress via increased levels of intracellular reactive oxygen species (ROS) [25,26], and both TH and ROS are reported to modulate RyR activity [27] and to influence axonal growth [28,29]. Therefore, in this study, we leveraged a primary rat hippocampal neuron-glia co-culture model to assess the relative contributions of TH and ROS-dependent mechanisms in mediating the axon inhibitory activity of BDE-49 and BDE-47. Our findings support the hypothesis that PBDEs inhibit axonal growth via ROS-dependent mechanisms, and that the TH, triiodothyronine (T3), protects against the effects of PBDEs on axonal growth by blocking PBDE-induced ROS.

## 2. Materials and Methods

### 2.1. Materials

Neat certified BDE-47 (2,2′,4,4′-tetrabromodiphenyl ether, >99% pure) and BDE-49 (2,2′,4,5′-tetrabromodiphenyl ether, >99% pure) were purchased from AccuStandard Inc. (New Haven, CT, USA), and verified for purity and composition by GC/MS by the UC Davis Superfund Research Program Analytical Core. Stock solutions of each BDE were made in dry dimethyl sulfoxide (DMSO, Sigma-Aldrich, St. Louis, MO, USA). 3,3′,5-Triiodo-L-thyronine (T3), N-acetyl-L-cysteine (NAC) and DL-α-tocopherol acetate were purchased from Sigma-Aldrich.

### 2.2. Animals

All procedures involving animals were approved by the University of California Davis Animal Care and Use Committee and conformed to the NIH Guide for the Care and Use of Laboratory Animals, and the ARRIVE guidelines [30]. Timed-pregnant Sprague Dawley rats were purchased from Charles River Laboratory (Hollister, CA, USA) and individually housed in clear plastic cages with corn cob bedding at 22 ± 2 °C under a 12 h dark–light cycle. Food and water were provided ad libitum.

### 2.3. Cell Culture

Primary neuron-glia co-cultures were prepared from hippocampi harvested from postnatal day (P) 0–1 male and female rat pups as previously described [31]. Briefly, rat pups were separated from the dam and anesthetized by placing them on a gauze pad on ice. Once pups ceased moving, they were euthanized by decapitation using sterile scissors. Hippocampi were harvested from the pup’s head by sterile dissection and then dissociated using trypsin (1 mg/mL) and DNAse (0.3 mg/mL). Dissociated hippocampal cells were plated on poly-L-lysine (0.5 mg/ML, Sigma Aldrich) coated glass coverslips (BellCo, Vineland, NJ, USA) and maintained at 37 °C in NeuralQ Basal Medium supplemented with 2% (*v*/*v*) GS21 (MTI-GlobalStem, Gaithersburg, MD, USA) and GlutaMAX (ThermoScientific, Waltham, MA, USA). The concentration of T3 in the complete medium used to maintain cultures was ~2.6 nM [32,33]. For studies of axonal growth, neurons were plated at 27,000 cells/cm^2^; for qPCR and Western blot experiments, neurons were plated at 105,000 cells/cm^2^. Cultures were exposed to varying concentrations of BDE-47 or BDE-49 diluted in culture medium from 1000× stocks; vehicle control cultures were exposed to DMSO (1:1000 dilution). A subset of cultures was co-exposed to T3, NAC, or α-tocopherol diluted 1:1000 directly into cell cultures from 1000× stocks in sterile distilled water.

### 2.4. Quantification of Axonal Outgrowth

Cultures were exposed to BDE-47, BDE-49, or vehicle (1:1000 DMSO) for 48 h beginning 3 h post-plating, and then fixed with 4% (*w*/*v*) paraformaldehyde (Sigma Aldrich) in 0.2 M phosphate buffer. To visualize axons, hippocampal cultures were immunostained with antibody specific for tau-1 (1:1000, Millipore, Billerica, MA, USA, RRID AB_94855). Our previous studies [23] demonstrated that exposure to BDE-47 or BDE-49 did not alter the expression of tau, as determined by Western blotting. Axonal lengths of tau-1 immunopositive neurons were manually quantified by an individual blinded to experimental condition using ImageJ software with the NeuronJ plugin [34]. As previously defined [35], in any given neuron, the axon was identified as the neurite whose length was >2.5× the cell body diameter and exceeded that of the other minor processes of the same neuron. Only non-overlapping neurons were quantified as proximity to other neurons can affect neuronal morphology.

### 2.5. Quantitative Polymerase Chain Reaction (qPCR)

Total RNA was isolated from cell cultures using TRIzol Reagent (ThermoScientific) per the manufacturer’s instructions, and cDNA was synthesized using the SuperScriptTMViloTM MasterMix containing SuperScriptTM III Reverse Transcriptase (Invitrogen, Carlsbad, CA, USA). Samples were mixed with Power SYBR Green MasterMix and forward and reverse primers (see Supplemental Table S1 for primer sequences and amplification efficiencies) and then loaded into a MicroAmp 384 Reaction Plate (ThermoScientific). qPCR plates were run on a 7900HT System by the Real-Time PCR Research and Diagnostics Core Facility at UC Davis. qPCR primers and probes were ordered from Integrated DNA Technologies (Coralville, IA, USA) using PrimeTime^®^ Predesigned qPCR Assays. Transcript levels were normalized to the average of the reference genes Ppia and Hprt1 and expression ratios were calculated by Pfaffl method [36] using REST 2009 software (Qiagen, Valencia, CA, USA).

### 2.6. ROS Measurements 

Rat hippocampal neurons cultures were exposed to BDE-47, BDE-49, or vehicle (1:1000 DMSO) in the absence or presence of T3, NAC, or α-tocopherol 3 h post-plating. Global ROS production was measured 1 h following exposures using ROS-Glo assay (Promega, Madison, WI, USA) according to manufacturer’s protocol, which specified using H_2_O_2_ as a positive technical control. Luminescence was recording using an H1 hybrid microplate reader (BioTek Instruments, Winooski, VT, USA).

### 2.7. Mitochondrial Metabolism Kinetics

Primary rat hippocampal neuron cultures were plated in 96-well plates at 27,000 cells/cm^2^ for 48 h. Cells were then exposed to BDE-47, BDE49, or vehicle (1:1000) in the absence or presence of T3 in combination with a mitochondrial substrate library, MitoPlate-S (Biolog, Inc., Hayward, CA, USA). Mitochondrial substrate metabolism was characterized according to the manufacturer’s protocol. Kinetics was recorded on the H1 hybrid microplate reader at a wavelength of 590 nm.

### 2.8. Statistics

All data are presented as mean ± SE unless otherwise indicated. Graphs were created in GraphPad Prism 8.3.0. Statistical analyses were performed with GraphPad Prism using one-way ANOVA with post hoc Tukey’s or Dunnett’s or post hoc Kruskal–Wallis with Dunn’s as appropriate for the normality of the data as measured by Shapiro–Wilk. qPCR data were analyzed using SDS 2.4 (ThermoScientific) and REST 2009 software (Qiagen, Valencia, CA, USA) with statistical analyses performed using REST 2009 pairwise reallocation randomization test. Significant differences between single and co-exposures or positive controls and vehicle were determined using Student’s *t*-test. Statistical significance was defined as *p* < 0.05.

## 3. Results

### 3.1. T3 Blocked the Axon Inhibitory Effects of BDE-47 and BDE-49

**Figure 1:**
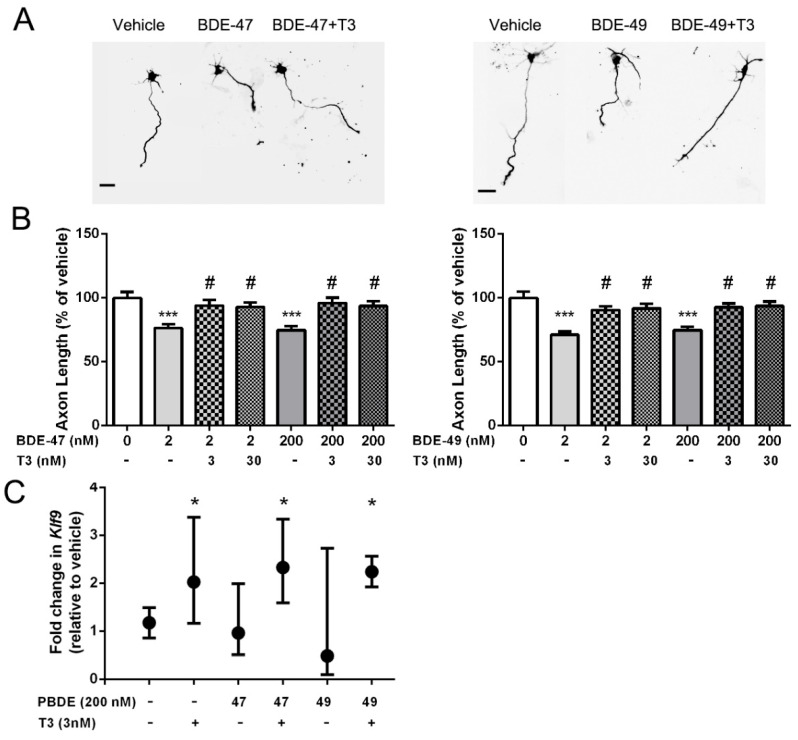
T3 supplementation prevented BDE-47 and BDE-49 inhibition of axonal growth in primary hippocampal neurons. Primary neuron-glia co-cultures dissociated from the hippocampi of P0-1 rats were exposed to vehicle (DMSO diluted 1:1000) or varying concentrations of BDE-47 or BDE-49 in the absence or presence of T3 beginning 3 h after plating. After 48 h exposure, cultures were fixed and immunostained for the axon-selective cytoskeletal protein tau-1. (**A**) Representative photomicrographs of DIV 2 hippocampal neurons exposed to vehicle, BDE 47 at 2 nM ± exogenous T3 at 3 nM. Scale bar = 25 µm. (**B**) Quantification of axon length in tau-1 immunopositive neurons. Data presented as the mean ± SE (*n* = 70–90 neurons from three independent dissections). *** Significantly different from vehicle at *p* < 0.001; # significantly different from the corresponding BDE treatment in the absence of T3 at *p* < 0.05 as determined by one-way ANOVA followed by Tukey’s post hoc test. (**C**) Fold-change in transcript levels of *Klf9* (as a % of vehicle control). Data are presented as the mean ± SE of *Klf9* expression normalized to the average of the reference genes *Ppia* and *Hprt1*. * Significantly different from vehicle at *p* < 0.05 as determined by REST 2009 pairwise randomization test.

We previously demonstrated that exposure to either BDE-47 or BDE-49 at concentrations ranging from 200 pM to 2 µM inhibited axonal growth in primary rat hippocampal neurons [23]. To address the question of whether these PBDE congeners modulated axonal growth via effects on TH signaling, we first tested whether the axon inhibitory activity of PBDEs could be blocked by supplementation of the culture medium with T3. Axon lengths were quantified on day in vitro (DIV) 2 after a 48 h exposure to BDE-47 or BDE-49 at 2 or 200 nM in the absence or presence of exogenous T3 at 3 or 30 nM. Consistent with our previous findings, BDE-47 or BDE-49 did not alter the number of axons extended by an individual neuron, but these PBDEs did significantly reduce axonal length relative to vehicle controls (Figure 1A,B). Addition of exogenous T3 at 3 or 30 nM, which raised T3 concentrations in the culture medium to ~5.6 and 32.6 nM, respectively, prevented the inhibition of axonal growth by BDE-47 or BDE-49, as indicated by the fact that axon lengths of neurons exposed to PBDEs in culture medium supplemented with T3 were not significantly different from those of vehicle controls (Figure 1A,B).

T3 is a component of many neuronal cell culture medias [34,35], and the medium used in these studies contained T3 at ~2.6 nM [34,35]. Thus, our observation that T3 supplementation protected against PBDE inhibition of axonal growth raised the possibility that PBDEs inhibited axonal growth by interfering with TH signaling. As one test of this possibility, we determined whether PBDEs interfered with TH-mediated gene expression. The gene Kruppel-like factor 9 (*Klf9*), previously known as Basic transcription element-binding protein (*Bteb*), has been shown to be a sensitive TH-responsive gene in the developing brain [36,37]. Analyses of *Klf9* transcripts in 2 DIV hippocampal cell cultures confirmed that *Klf9* expression is significantly upregulated in cultures exposed to exogenous T3 at 3 nM for 48 h (Figure 1C). In contrast, exposure to BDE-47 or BDE-49 at 200 nM for 48 h had no significant effect on *Klf9* transcript levels relative to vehicle control cultures and did not inhibit the upregulation of *Klf9* by T3 (Figure 1C).

**Figure 2:**
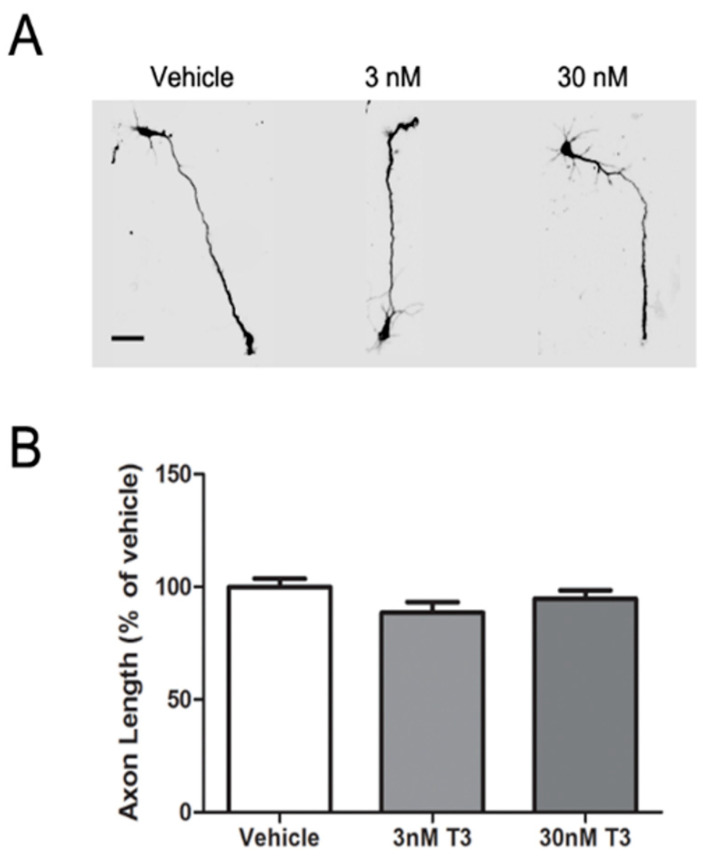
T3 did not influence axonal growth. Primary neuron-glia co-cultures dissociated from the hippocampi of P0-1 rat hippocampi were exposed to vehicle (DMSO diluted 1:1000) or T3 and/or BDE-47 or BDE-49 beginning 3 h after plating. After 48 h exposure, cultures were fixed and immunostained for tau-1. Representative photomicrographs (**A**) and quantification of axon length (**B**) in tau-1 immunopositive neurons at DIV 2. Data are presented as the mean ± SE (*n* = 30–40 neurons per group from one dissection; results repeated in 3 independent dissections). There were no significant differences between neurons exposed to vehicle vs. T3 as determined by one-way ANOVA (*p* < 0.05). Scale bar = 25 µm.

To determine whether the protective effect of T3 on PBDE inhibition of axonal growth was mediated via direct effects of T3 on axonal growth, we quantified the effect of supplementing the culture medium with T3 on axonal growth in cultures not exposed to PBDEs. As seen in representative photomicrographs (Figure 2A), supplementation with T3 at either 3 or 30 nM had no obvious effect on axonal morphology in terms of the number, length, or branching of axons in DIV 2 hippocampal neurons. Quantification of axon length confirmed that 48 h exposure to medium supplemented with T3 did not significantly alter axon length relative to that observed in vehicle control cultures (Figure 2B).

### 3.2. Antioxidants Blocked PBDE Inhibition of Axonal Growth

**Figure 3:**
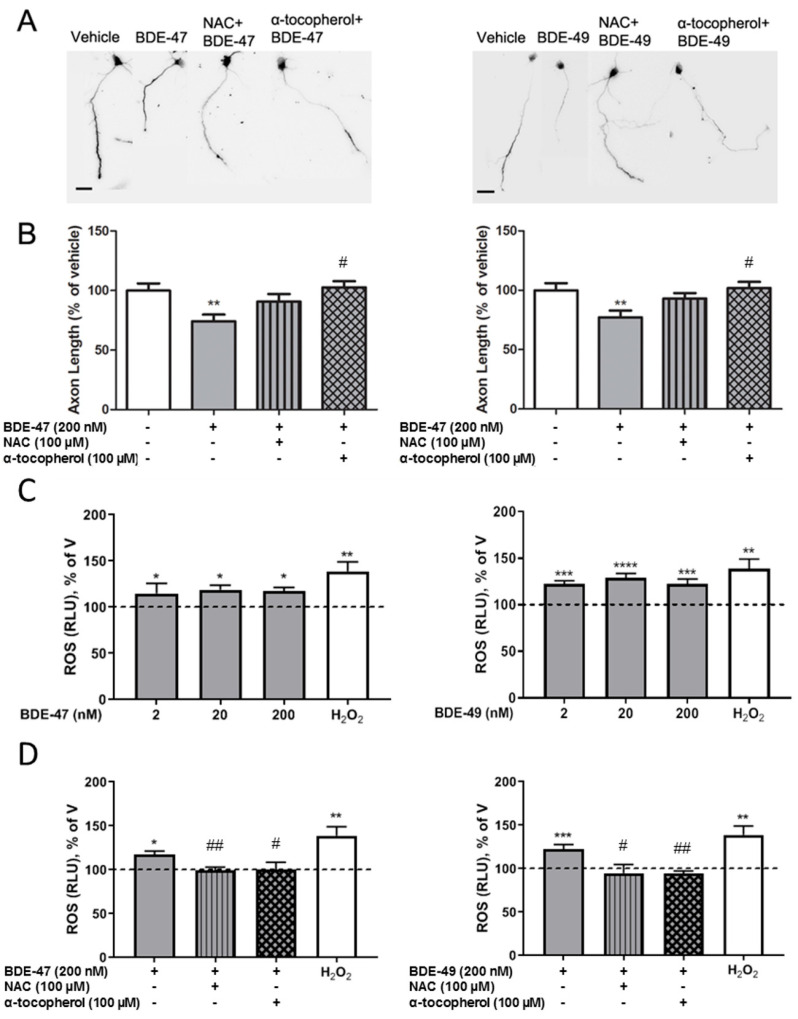
Antioxidants prevented BDE-47 and BDE-49 inhibition of axonal growth and production of ROS. Primary neuron-glia co-cultures dissociated from the hippocampi of P0-1 rat pups were exposed to vehicle, BDE-47 or BDE-49 in the absence or presence of N-acetyl cysteine (NAC) or α-tocopherol. After 48 h exposure, cultures were fixed and immunostained for tau-1. (**A**) Representative photomicrographs of DIV 2 hippocampal neurons from different experimental groups. Scale bar = 25 µm. (**B**) Quantification of axon length in tau-1 immunopositive cells (*n* = 70–90 neurons from three independent dissections). Quantification of ROS levels following exposure to vehicle, BDE-47 or BDE-49 alone (**C**) or in the presence of an antioxidant (**D**) (*n* = three independent dissections). H_2_O_2_ was included as a positive technical control for the ROS-Glo assay per the manufacturer’s instructions. Data presented as the mean ± SE. * Significantly different from vehicle at * *p* < 0.05, ** *p* < 0.01, *** *p* < 0.001, **** *p* < 0.0001; # significantly different from PBDE treatment alone at # *p* < 0.05, ## *p* < 0.01, as determined by one-way ANOVA followed by Tukey’s post hoc test; †significantly different from individual PBDE treatment at *p* < 0.05 as determined by Student’s *t*-test.

Previous reports have demonstrated that PBDEs increase levels of ROS in cultured neurons [38,39,40] and that PBDE-induced ROS can be blocked by mechanistically diverse antioxidants, specifically the NADPH oxidase inhibitor, NAC, or the ROS scavenger, α-tocopherol [41,42]. To evaluate a role for ROS in the axon inhibitory effects of PBDEs, we thus determined whether co-exposure to NAC or α-tocopherol blocked the inhibition of axonal growth by BDE-47 or BDE-49. No significant changes in axon length were observed with antioxidant treatment alone (Supplemental material Figure S1). As shown in representative photomicrographs (Figure 3A) and confirmed by quantitative morphometric analyses of axons (Figure 3B), axon lengths of hippocampal neurons exposed to BDE-47 or BDE-49 at 200 nM in the presence of 100 µM NAC or 100 µM α-tocopherol were not significantly different from those of vehicle control neurons. Cultures co-exposed to PBDEs and antioxidants were significantly longer than axon lengths of hippocampal neurons exposed to the corresponding BDE alone.

To determine whether nM concentrations of BDE-47 or BDE-49 that inhibit axonal growth increased intracellular ROS, ROS were measured in cultures acutely exposed to BDE-47 or BDE-49. Both BDE-47 and BDE-49-exposed cultures had higher amounts of ROS compared to vehicle control cultures (Figure 3C). We next evaluated whether antioxidants blocked the inhibitory effects of PBDEs on axonal growth by providing protection against ROS generation (Figure 3D). In the presence of NAC or α-tocopherol, PBDEs did not produce significant amounts of ROS compared to vehicle. However, ROS production was substantially reduced compared to BDE-47 or BDE-49 alone.

**Figure 4:**
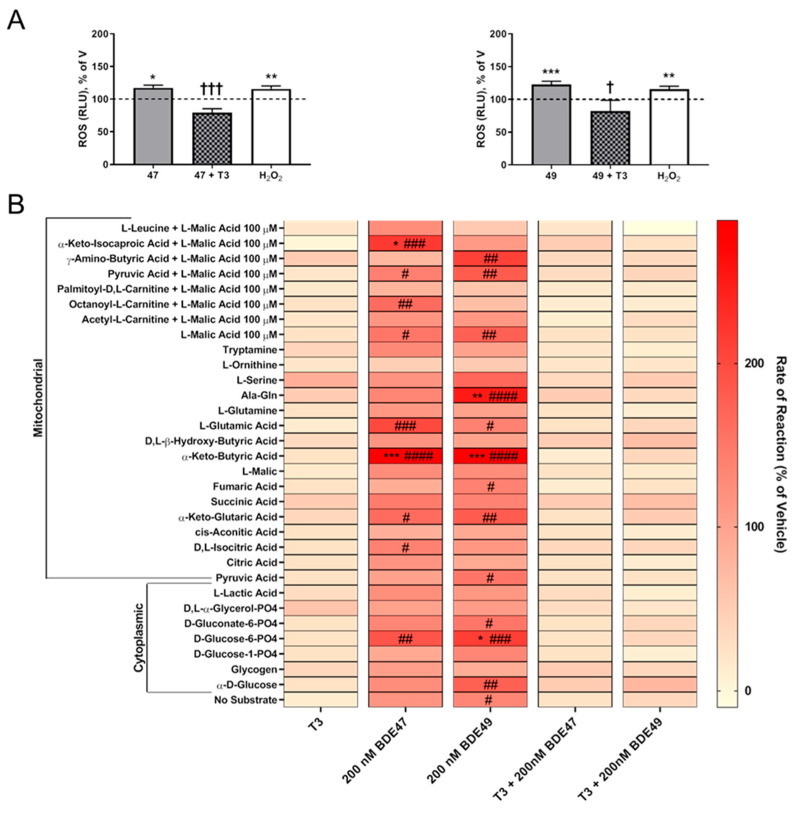
T3 normalized ROS levels and mitochondrial substrate metabolism in cultures exposed to BDE-47 or BDE-49. Hippocampal neuron-glia co-cultures were exposed to vehicle, T3, BDE-47 and/or BDE-49 for 1 h on DIV 2. (**A**) Quantification of ROS production following co-exposure to T3 and PBDEs. (**B**) Mitochondrial substrate metabolism kinetics immediately following PBDE exposure alone and in the presence of T3. Data presented as the mean ± SE (*n* = three independent dissections). * Significantly different from vehicle at * *p* < 0.05, ** *p* < 0.01, *** *p* < 0.001; # significantly different from T3 at # *p* < 0.05, ## *p* < 0.01, ### *p* < 0.001, #### *p* < 0.0001 as determined by one-way ANOVA followed by Dunnett’s post hoc test; †significantly different from individual PBDE treatment at † *p* < 0.05, ††† *p* < 0.001 as determined by Student’s *t*-test.

It is posited that ROS generation largely originates from mitochondrial damage [43]. BDE-47 can disrupt the mitochondrial membrane potential [44], while both BDE-47 [45,46] and BDE-49 [47] can decrease mitochondrial bioenergetics. Thus, we next sought to determine whether acute exposure to nM concentrations of BDE-47 or BDE-49 altered mitochondrial metabolism. Compared to vehicle control cultures, mitochondrial metabolism was significantly impacted in cultures acutely exposed to either BDE-47 or BDE-49 at 200 nM (Figure 4B).

### 3.3. T3 Blocked PBDE Axon Inhibition by Blocking PBDE-Induced ROS

To determine whether T3 conferred protection against the axon inhibitory effects of PBDEs via upregulation of endogenous antioxidant molecules, we quantified the effects of T3, BDE-47, and BDE-49, alone and in combination, on the production of ROS (Figure 4A). In contrast to cultures exposed to PBDEs in the absence of T3, in cultures co-exposed for 1 h to one of these PBDEs and T3 exhibited no significant change in ROS levels relative to vehicle controls. Moreover, ROS levels were significantly reduced in cultures co-exposed to PBDEs and T3 relative to cultures exposed to PBDEs in the absence of T3. We then explored whether T3 protected against disrupted mitochondrial bioenergetics (Figure 4B). Following acute exposure to either BDE-47 or BDE-49 in combination with T3, there were no marked alterations in mitochondrial substrate metabolism relative to vehicle. In addition, any effects observed with individual PBDE exposure were eliminated in cultures co-exposed to PBDEs and T3.

## 4. Discussion

The findings from this study extend our previous report that BDE-47 and BDE-49 inhibited axonal growth in primary rat hippocampal neurons [23] by demonstrating that the axon inhibitory activity of these PBDE congeners is mediated by increased levels of intracellular ROS. The evidence in support of this conclusion includes: (1) BDE-47 and BDE-49 increased ROS in primary rat hippocampal neurons at nM concentrations that also inhibited axonal growth; and (2) co-exposure to either the NADPH oxidase inhibitor, NAC, or the ROS scavenger, α-tocopherol, blocked the axon inhibitory effects of BDE-47 and BDE-49. Additionally, we observed that supplementation of the culture medium with exogenous T3 blocked the inhibition of axonal growth in PBDE-exposed neuronal cultures, coincident with mitigation of PBDE effects on intracellular ROS and metabolic substrate production from the mitochondria. These findings suggest a role for T3 in maintaining intracellular redox homeostasis in response to pro-oxidants, which if true, represents a novel mechanism by which thyroid hormone disruption contributes to adverse neurodevelopmental outcomes. 

Our observations are consistent with previous reports that PBDEs upregulated biomarkers of oxidative stress in the brain of adult and developing rodent models [48,49] and increased intracellular ROS levels in cultured neural cells [38,39,50,51]. This earlier work demonstrated that µM concentrations of PBDEs increased intracellular ROS in cultured neurons to levels that triggered apoptosis [24,52,53]. Here, we found that exposure of primary hippocampal neuron-glia co-cultures to BDE-47 or BDE-49 at nM concentrations also increased intracellular ROS, but this was associated with inhibited axonal growth. Our data extend reports in the literature indicating that physiologic levels of ROS regulate axonal specification and axonal growth in primary hippocampal neurons, and modulation of ROS synthesis in axonal growth cones cause cytoskeletal rearrangements that alter axonal morphogenesis [54]. Collectively, these observations suggest a model in which nM PBDE concentrations increase ROS locally in the axonal growth cone to modulate signaling pathways that regulate axonal growth [55,56], whereas µM PBDE concentrations increase intracellular ROS globally to trigger cell death. Confirmation of this model will require the adaptation of sensitive technologies to detect localized changes in ROS in subcellular domains of neurons [57] exposed to PBDEs at concentrations that inhibit axonal growth. 

PBDEs can interfere with thyroid hormone signaling and thyroid hormone disruption is widely posited to contribute to the developmental neurotoxicity of these environmental contaminants [25,26]. PBDEs have been shown to suppress dendritic growth in Purkinje cells by disrupting TH receptor-mediated transcription [58], and we observed that co-exposure to T3 blocked inhibition of axonal growth by BDE-47 or BDE-49. However, several lines of evidence argue against the hypothesis that PBDEs inhibit axonal growth in hippocampal neurons via direct interference with TH signaling. First, in hippocampal cultures not exposed to PBDEs, T3 supplementation of the culture medium did not promote axonal growth. Second, exposure of hippocampal cultures to BDE-47 or BDE-49 did not alter expression of *Klf9*, a gene known to be highly sensitive to upregulation by TH in the developing brain [59]. Nor did BDE-47 or BDE-49 significantly block T3-induced *Klf9* expression. These findings are in agreement with previous studies [58] in which qPCR analyses detected no significant changes in transcript levels of TH-responsive genes, including TRα1 or TRβ, in primary rat Purkinje cells exposed to PBDEs. Moreover, since the affinity of T3 to the thyroid hormone receptor (THR) is approximately 0.1 nM, the observation that T3 present in the medium without addition of extra T3 is not sufficient to prevent the axon inhibitory effects of PBDEs suggests that the neuroprotective effect of exogenous T3 in this model is mediated by THR-independent mechanisms. NH-3, a pharmacological THR modulator with mixed agonist/antagonistic activity [60], may be useful for addressing this question, but given experimental evidence that the concentration–response relationship for antagonistic vs. agonist effects of NH-3 vary across models, its effectiveness in mechanistic studies of the axon inhibitory activity of PBDEs will require identification of a concentration that antagonizes THR in this model system [60,61].

Our data suggest that T3 supplementation prevented PBDE inhibition of axonal growth by mitigating PBDE-induced ROS. Specifically, we observed that T3 supplementation ameliorated PBDE-induced ROS generation. A key question is how. TH has been reported to upregulate expression of endogenous antioxidant molecules [62,63]. However, preliminary qPCR analyses failed to detect significant upregulation of several endogenous antioxidants in primary hippocampal neuron-glia co-cultures exposed to BDE-47 or BDE-49 in the presence of T3 (Supplemental Table S2). This observation does not rule out the possibility that T3 upregulated expression of cellular antioxidants other than those we assessed and/or that T3 increased the activity of enzymatic antioxidants. In addition, *Klf9* upregulation by 5 or 10 nM T3 supplementation has previously been shown to protect the axons of primary cortical murine neurons from hypoxic injury [64]. Whether *Klf9* or other T3-regulated targets are directly involved in mitigating PBDE axon inhibition remains to be investigated. However, it is now clear that TH can also signal via non-transcriptional mechanisms [65,66,67], including direct influence on mitochondrial respiration [68]. Consistent with this literature, our data support a model in which T3 protects mitochondrial metabolism against PBDE-mediated disruption of mitochondrial bioenergetics.

The observation that T3 prevented PBDE-induced changes in mitochondrial substrate utilization at concentrations that also blocked PBDE inhibition of axon growth, yet had no effect on basal axonogenesis, suggested that PBDEs increased ROS as a consequence of altered mitochondrial metabolism. In support of this proposed mechanism, at concentrations that increased intracellular ROS levels, BDE-47 and BDE-49 increased utilization of metabolic substrates (α-keto-isocaproic acid, α-keto-butyric acid, Ala-Gln, D-glucose-6-PO4) used to produce NADH, and disruption of NADH production has been linked to increased ROS generation [69]. However, the mechanism(s) by which PBDEs interfere with mitochondrial metabolism remain to be elucidated.

Findings from our previous studies suggested RyR was a downstream effector in PBDE-induced axon growth inhibition [23]. Given the redox-sensitive nature of RyR [70,71] and spatial relationship with mitochondria [72], a potential indirect mechanism presents itself wherein mitochondrial ROS production alters RyR gating and, consequently, calcium signaling to interfere with axon growth. This model is supported by experimental evidence demonstrating that disruption of mitochondrial function affects calcium homeostasis, which in turn delays polarization of developing neurons and inhibits axonal growth [73]. As we previously reported [23], PBDE inhibition of axonal growth is due in part to delayed neuronal polarization. The role of RyR as a downstream key event rather than the molecular initiating event in PBDE developmental neurotoxicity may explain the differential response of dendrites vs. axons to non-dioxin-like polychlorinated biphenyls (PCBs) vs. PBDEs. Specifically, in primary rat hippocampal and cortical neuron-glia co-cultures, non-dioxin-like PCBs were observed to promote dendritic growth, but have no effect on axonal growth, and the dendrite promoting activity was mediated by RyR sensitization [74,75]. In contrast, PBDEs were observed to inhibit axonal growth but have no effect on dendritic growth in the same neuronal cell culture model [23].

In summary, our study provides novel insight into the interplay between ROS, TH, and axonal growth in PBDE developmental neurotoxicity. Whether PBDE interference with axonal growth contributes to adverse neurodevelopmental outcomes in vivo is still to be determined; however, clinical [20,21] and experimental evidence [17,18,19] demonstrate that altered spatiotemporal patterns of axonal growth during brain development can cause functional deficits. Susceptibility to this neurotoxic activity of PBDEs may be enhanced in populations with heritable mutations that alter mitochondrial and redox signaling, which are themselves associated with increased risk of neurodevelopmental disorders [76,77]. The finding that T3 protects against axon growth inhibition by BDE-47 and BDE-49 in vitro suggests that PBDE-mediated TH dysregulation [2,78] also has the potential to amplify PBDE effects on axonal growth in vivo. Further studies into gene × environment interactions associated with these mechanisms may lead to a better understanding of populations with increased vulnerability to PBDE developmental neurotoxicity.

## Data Availability

Data will be made available upon reasonable request.

## Supplementary Materials

The following supporting information can be downloaded at: https://www.mdpi.com/article/10.3390/toxics10020092/s1, Figure S1: The antioxidants NAC and α-tocopherol do not alter axonal outgrowth relative to vehicle control cultures. Primary neuron-glia co-cultures dissociated from the hippocampi of P0-1 rat pups were exposed to vehicle, N-acetyl cysteine (NAC) or α-tocopherol. After a 48 h exposure, cultures were fixed and immunostained for tau-1. Axon length was quantified in tau-1 immunopositive cells (*n* = 70–90 neurons from three independent dissections). Data presented as the mean ± SE. No significant differences between groups was detected using one-way ANOVA (*p* < 0.05); Table S1: Primer sequences and amplification efficiencies; Table S2: Average fold changes relative to vehicle of levels of transcripts encoding cellular antioxidants.
