# Supplementary material for: Triiodothyronine or Antioxidants Block the Inhibitory Effects of BDE-47 and BDE-49 on Axonal Growth in Rat Hippocampal Neuron-Glia Co-Cultures
Source: Toxics. 2022 Feb 18;10(2):92. doi: 10.3390/toxics10020092 (PMC8879960; doi:10.3390/toxics10020092)
Supplement: Supplementary file 1 [file toxics-10-00092-s001.zip › toxics-1546675-supplementary.pdf]

toxics

MDPI

Article

Supplementary Materials: Triiodothyronine or Antioxidants Block the Inhibitory Effects of BDE-47 and BDE-49 on Axonal Growth in Rat Hippocampal Neuron-Glia Co-Cultures

Hao Chen, Rhianna K. Carty, Adrienne C. Bautista, Keri A. Hayakawa and Pamela J. Lein

Table S1. Primer sequences and amplification efficiencies.

Gene	Primer 1 (5′-3′)	Primer 2 (5′-3′)	Amplification Efficiency (%)
Klf9	CACCGAATCTGGGTCGAGTC	CCGTTCACCTGTATGCACTC	100
Cat	*CGCTGAACAAGAAAGTAACCTG*	*CCATCCTTTATCCATAGCCAGAAG*	100
Nos2	*TGTCTGTGACTTTGTGCTTCT*	*GACTGGACTTTTAGAGACGCTT*	100
Gclc	*GCCGCCATTCAGTAACAAC*	*ACATCTACCACGCAGTCAAG*	100
Gpx1	*CCATTCACCTCGCACTTCT*	*AATCAGTTCGGACATCAGGAG*	100
Gpx4	*CGCAGCCGTTCTTATCAATG*	*CACTGTGGAAATGGATGAAAGTC*	100
Sod1	*GCCTTGTGTATTGTCCCCATA*	*CGTCATTCACTTCGAGCAGA*	100
Sod2	*ATTGAACTTCAGTGCAGGCT*	*CGACCTACGTGAACAATCTGA*	100
Nrf2	*CAAGCGACTCATGGTCATCTAC*	*CAGTGGATCTGTCAGCTACTC*	100
Ppia	*TTTGCAGACGCCGCTGT*	*ATCAGCCGTGATGTCGAAG*	100
Hprt1	*GGTGAAAAGGACCTCTCGAAG*	*GCTTTTCCACTTTCGCTGATG*	100

Table S2. Average fold changes relative to vehicle of levels of transcripts encoding cellular antioxidants.

Gene	T3 (3 nM)	BDE-47 (200 nM)	BDE-49 (200 nM)	BDE-47 + T3	BDE-49 + T3
Cat	0.994	0.758	0.552	0.409	0.513
Nos2	1.003	0.888	0.236	0.176	***0.107*** *
Gclc	0.917	1.006	0.738	0.491	***0.425*** *
Gpx1	0.811	1.331	0.967	0.864	0.761
Gpx4	0.592	0.979	0.675	0.468	0.469
Sod1	1.096	1.473	0.813	1.535	1.171
Sod2	1.28	1.596	0.858	1.686	1.32
Nrf2	1.421	0.752	0.581	1.135	1.196

Notes: Data presented as the mean ($n = 3$ independent dissections). Hippocampal cultures from PND 0-1 rat pups were treated with BDE-47, BDE-49 or vehicle (1:1000 DMSO) in the absence or presence of T3 for 48 h beginning at 3h post-plating. Fold changes in expression relative to vehicle treated cultures were calculated using REST 2009 software. * Significantly different from vehicle at $p < 0.05$ as determined by REST 2009 pairwise reallocation randomization test.

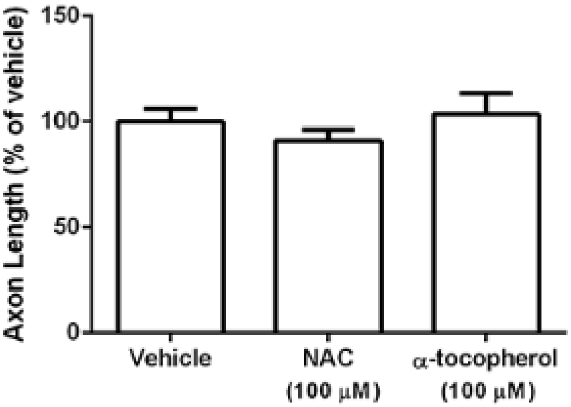

Figure S1. The antioxidants NAC and α-tocopherol do not alter axonal outgrowth relative to vehicle control cultures. Primary neuron-glia co-cultures dissociated from the hippocampi of P0-1 rat pups were exposed to vehicle, N-acetyl cysteine (NAC) or α-tocopherol. After a 48 h exposure, cultures were fixed and immunostained for tau-1. Axon length was quantified in tau-1 immunopositive cells (n = 70 – 90 neurons from three independent dissections). Data presented as the mean ± SE. No significant differences between groups was detected using one-way ANOVA ($p < 0.05$).
